# Supplementary material for: Spatiotemporal modulation of a common set of muscle synergies during unpredictable and predictable gait perturbations in older adults
Source: J Exp Biol. 2024 Apr 11;227(7):jeb247271. doi: 10.1242/jeb.247271 (PMC11058090; doi:10.1242/jeb.247271)
Supplement: Supplementary information [file jexbio-227-247271-s1.pdf]

Table S1. Results of the linear mixed-effects model for the investigated parameters of muscle synergies and gait. For each effect the degrees of freedom between conditions (DF_num) and within condition (DF_denom), the F-value and the p-value are presented. Conditions are unpredictable and predictable trials. CS=cosine similarity, CoA = center of activity, FWHM = full width at half maximum.

		effect	DF_num	DF_denom	CS of activation patterns		CS of muscle weights	
					F-value	p-value	F-value	p-value
anteroposterior	Weight acceptance	(Intercept)	1	982	12546.6	< 0.001	6535.1	< 0.001
		condition	1	982	17.6	< 0.001	9.7	0.002
		cycles	10	982	69.2	< 0.001	16.5	< 0.001
		condition:cycles	10	982	17.6	< 0.001	3.8	< 0.001
	Propulsion	(Intercept)	1	1164	14638.1	< 0.001	7309.6	< 0.001
		condition	1	1164	62.1	< 0.001	6.9	0.009
		cycles	10	1164	47.3	< 0.001	2.4	0.007
		condition:cycles	10	1164	5.2	< 0.001	0.8	0.583
	Early swing	(Intercept)	1	797	21585.6	< 0.001	26930.3	< 0.001
		condition	1	797	50.6	< 0.001	6.3	0.012
		cycles	10	797	56.1	< 0.001	21.1	< 0.001
		condition:cycles	10	797	5.1	< 0.001	1	0.473
	Late swing	(Intercept)	1	1042	20618.2	< 0.001	15466.6	< 0.001
		condition	1	1042	36.5	< 0.001	6.8	0.009
		cycles	10	1042	33.1	< 0.001	29.1	< 0.001
		condition:cycles	10	1042	5.4	< 0.001	1.8	0.062
mediolateral	Weight acceptance	(Intercept)	1	912	7887.5	< 0.001	3542.1	< 0.001
		condition	1	912	6.5	0.011	2.5	0.115
		cycles	10	912	22.4	< 0.001	6.1	< 0.001
		condition:cycles	10	912	5.3	< 0.001	4.2	< 0.001
	Propulsion	(Intercept)	1	1038	12543.6	< 0.001	6955.8	< 0.001
		condition	1	1038	211.2	< 0.001	80.4	< 0.001
		cycles	10	1038	40.2	< 0.001	9.5	< 0.001
		condition:cycles	10	1038	5.6	< 0.001	4.3	< 0.001
	Early swing	(Intercept)	1	674	13366.6	< 0.001	17307.4	< 0.001
		condition	1	674	23	< 0.001	16.1	< 0.001
		cycles	10	674	37.4	< 0.001	23.2	< 0.001
		condition:cycles	10	674	5.9	< 0.001	7.6	< 0.001
	Late swing	(Intercept)	1	832	16486.3	< 0.001	19436.8	< 0.001
		condition	1	832	42.5	< 0.001	4.2	0.042
		cycles	10	832	61.4	< 0.001	24.2	< 0.001
		condition:cycles	10	832	6.7	< 0.001	6.9	< 0.001

Journal of Experimental Biology • Supplementary information

		effect	DF_num	DF_denom	CoA		FWHM	
					F-value	p-value	F-value	p-value
anteroposterior	Weight acceptance	(Intercept)	1	1252	739.9	< 0.001	1490.6	< 0.001
		condition	1	1252	54.1	< 0.001	1.6	0.199
		cycles	11	1252	70.6	< 0.001	20.6	< 0.001
		condition:cycles	11	1252	24.9	< 0.001	2.7	0.002
	Propulsion	(Intercept)	1	1332	9231.1	< 0.001	1459.8	< 0.001
		condition	1	1332	29.1	< 0.001	0.6	0.436
		cycles	11	1332	2.2	0.011	2.5	0.004
		condition:cycles	11	1332	3.9	< 0.001	2.5	0.004
	Early swing	(Intercept)	1	1053	11439.2	< 0.001	580.6	< 0.001
		condition	1	1053	0.9	0.349	4.9	0.027
		cycles	11	1053	36.7	< 0.001	5.4	< 0.001
		condition:cycles	11	1053	5.1	< 0.001	1.7	0.076
	Late swing	(Intercept)	1	1234	1485.4	< 0.001	2238.7	< 0.001
		condition	1	1234	11.3	0.001	0.3	0.579
		cycles	11	1234	3.2	< 0.001	4.6	< 0.001
		condition:cycles	11	1234	1.3	0.227	1.1	0.346
mediolateral	Weight acceptance	(Intercept)	1	1177	552.4	< 0.001	1651	< 0.001
		condition	1	1177	30.6	< 0.001	0.2	0.682
		cycles	11	1177	57	< 0.001	17.1	< 0.001
		condition:cycles	11	1177	12.7	< 0.001	2.6	0.003
	Propulsion	(Intercept)	1	1268	8177.3	< 0.001	1397	< 0.001
		condition	1	1268	77.7	< 0.001	0.8	0.358
		cycles	11	1268	31	< 0.001	9.7	< 0.001
		condition:cycles	11	1268	6.5	< 0.001	3.8	< 0.001
	Early swing	(Intercept)	1	964	11234.3	< 0.001	676.6	< 0.001
		condition	1	964	10.5	0.001	1.8	0.179
		cycles	11	964	21.4	< 0.001	1.3	0.239
		condition:cycles	11	964	6.2	< 0.001	1.1	0.340
	Late swing	(Intercept)	1	1089	1246	< 0.001	1937.9	< 0.001
		condition	1	1089	6.5	0.011	0.5	0.499
		cycles	11	1089	6.9	< 0.001	10.5	< 0.001
		condition:cycles	11	1089	0.7	0.713	3.6	< 0.001

Journal of Experimental Biology • Supplementary information

		effect	DF_num	DF_denom	Gait parameter	
					F-value	p-value
anteroposterior	Cadence	(Intercept)	1	1366	11963.9	< 0.001
		condition	1	1366	77.9	< 0.001
		cycles	11	1366	95.4	< 0.001
		condition:cycles	11	1366	23.6	< 0.001
	Stance time	(Intercept)	1	1366	9855.4	< 0.001
		condition	1	1366	93.5	< 0.001
		cycles	11	1366	98.5	< 0.001
		condition:cycles	11	1366	22.3	< 0.001
	Swing time	(Intercept)	1	1366	15365.1	< 0.001
		condition	1	1366	55.5	< 0.001
		cycles	11	1366	172.7	< 0.001
		condition:cycles	11	1366	23.2	< 0.001
	Duty factor	(Intercept)	1	1366	99892.6	< 0.001
		condition	1	1366	12.2	< 0.001
		cycles	11	1366	55.5	< 0.001
		condition:cycles	11	1366	10.4	< 0.001
mediolateral	Cadence	(Intercept)	1	1294	11217.5	< 0.001
		condition	1	1294	277.6	< 0.001
		cycles	11	1294	110.4	< 0.001
		condition:cycles	11	1294	56.5	< 0.001
	Stance time	(Intercept)	1	1294	9973	< 0.001
		condition	1	1294	341.7	< 0.001
		cycles	11	1294	152.4	< 0.001
		condition:cycles	11	1294	68	< 0.001
	Swing time	(Intercept)	1	1294	12629.2	< 0.001
		condition	1	1294	115.7	< 0.001
		cycles	11	1294	132.5	< 0.001
		condition:cycles	11	1294	26.3	< 0.001
	Duty factor	(Intercept)	1	1294	104383.1	< 0.001
		condition	1	1294	22.3	< 0.001
		cycles	11	1294	135.3	< 0.001
		condition:cycles	11	1294	34.3	< 0.001

Table S2. Post-hoc results of the linear mixed-effect models for all parameters of muscle synergies and gait. For all pairwise compared cycles p-values and effect sizes (Cohen's d) are presented. Cycles include the three unperturbed cycles (-3 to -1), the perturbation cycle (0) and the following 8 recovery cycles (1 to 8).

Cosine similarity of the activation patterns

	compared cycles	anteroposterior								mediolateral							
		Weight acceptance		Propulsion		Early swing		Late swing		Weight acceptance		Propulsion		Early swing		Late swing	
		p-value	*d*	p-value	*d*	p-value	*d*	p-value	*d*	p-value	*d*	p-value	*d*	p-value	*d*	p-value	*d*
unpredictable	-3 vs. -2	-	-	-	-	-	-	-	-	-	-	-	-	-	-	-	-
	-3 vs. -1	0.767	0.12	0.411	-0.21	0.678	0.15	0.610	-0.09	0.742	0.13	0.630	-0.10	0.789	-0.13	0.930	0.03
	-3 vs. 0	0.000	4.10	0.000	1.83	0.000	2.61	0.000	1.56	0.000	2.27	0.000	1.62	0.000	2.17	0.000	2.95
	-3 vs. 1	0.000	1.54	0.000	1.92	0.000	2.95	0.000	2.14	0.000	1.26	0.000	1.97	0.000	2.17	0.000	2.61
	-3 vs. 2	0.016	0.54	0.000	0.71	0.000	0.99	0.000	0.70	0.011	0.58	0.000	1.43	0.000	0.98	0.000	1.37
	-3 vs. 3	0.313	0.30	0.014	0.46	0.021	0.62	0.168	0.30	0.081	0.45	0.000	0.82	0.002	0.75	0.001	0.73
	-3 vs. 4	0.043	0.48	0.411	0.21	0.289	0.34	0.168	0.30	0.332	0.29	0.004	0.53	0.704	0.19	0.135	0.36
	-3 vs. 5	0.619	0.19	0.745	0.07	0.171	0.41	0.504	0.12	0.126	0.38	0.614	0.11	0.412	0.29	0.098	0.40
	-3 vs. 6	0.948	-0.03	0.411	-0.20	0.704	-0.12	0.413	-0.17	0.353	-0.27	0.321	-0.20	0.412	-0.30	0.493	-0.20
	-3 vs. 7	0.923	-0.08	0.561	-0.13	0.226	-0.38	0.299	-0.23	0.693	-0.16	0.048	-0.37	0.706	-0.17	0.271	-0.28
	-3 vs. 8	0.182	-0.35	0.700	0.09	0.165	-0.42	0.161	-0.33	0.742	-0.15	0.321	0.20	0.906	0.07	0.921	-0.06
predictable	-3 vs. -2	-	-	-	-	-	-	-	-	-	-	-	-	-	-	-	-
	-3 vs. -1	0.827	-0.09	0.557	0.14	0.704	0.09	0.161	0.31	0.951	0.01	0.965	-0.01	0.939	-0.02	0.832	0.11
	-3 vs. 0	0.000	1.26	0.000	1.20	0.000	1.46	0.000	0.87	0.000	0.89	0.000	1.06	0.000	1.64	0.000	1.82
	-3 vs. 1	0.000	1.23	0.000	0.98	0.000	1.55	0.000	1.05	0.126	0.36	0.000	0.80	0.218	0.40	0.000	1.31
	-3 vs. 2	0.948	-0.01	0.477	0.16	0.440	0.21	0.077	0.39	0.743	0.10	0.000	0.63	0.704	0.17	0.492	0.20
	-3 vs. 3	0.713	-0.14	0.955	0.02	0.704	0.09	0.168	0.29	0.081	0.40	0.092	0.32	0.566	0.25	0.930	-0.03
	-3 vs. 4	0.948	0.03	0.948	0.03	0.704	0.08	0.476	0.14	0.693	0.16	0.209	0.24	0.939	-0.03	0.921	-0.06
	-3 vs. 5	0.948	0.03	0.955	0.02	0.421	0.22	0.360	0.20	0.353	0.24	0.908	0.04	0.906	-0.07	0.982	0.00
	-3 vs. 6	0.948	-0.03	0.660	-0.10	0.704	0.10	0.400	-0.18	0.742	-0.13	0.986	0.00	0.939	0.03	0.930	-0.03
	-3 vs. 7	0.619	-0.17	0.561	-0.13	0.704	-0.09	0.400	-0.18	0.927	-0.04	0.348	-0.18	0.906	-0.07	0.930	0.04
	-3 vs. 8	0.923	-0.06	0.960	-0.01	0.261	-0.30	0.204	-0.27	0.742	0.11	0.965	0.02	0.704	0.16	0.630	-0.15
unpredictable vs. predictable	-3 vs. -3	-	-	-	-	-	-	-	-	-	-	-	-	-	-	-	-
	-2 vs. -2	0.619	-0.17	0.426	0.18	0.989	0.00	0.415	0.16	0.693	-0.16	0.011	0.47	0.822	-0.12	0.982	-0.01
	-1 vs. -1	0.948	0.04	0.477	-0.17	0.806	0.06	0.262	-0.25	0.927	-0.05	0.043	0.38	0.614	-0.22	0.887	-0.08
	0 vs. 0	0.000	2.67	0.000	0.81	0.000	1.15	0.000	0.85	0.000	1.22	0.000	1.03	0.188	0.41	0.000	1.12
	1 vs. 1	0.737	0.13	0.000	1.13	0.000	1.40	0.000	1.24	0.000	0.73	0.000	1.63	0.000	1.65	0.000	1.29
	2 vs. 2	0.118	0.38	0.000	0.73	0.001	0.78	0.018	0.48	0.236	0.31	0.000	1.27	0.005	0.70	0.000	1.15
	3 vs. 3	0.337	0.27	0.000	0.62	0.035	0.52	0.414	0.17	0.742	-0.12	0.000	0.97	0.216	0.38	0.000	0.76
	4 vs. 4	0.328	0.28	0.073	0.36	0.404	0.26	0.161	0.32	0.927	-0.04	0.000	0.75	0.861	0.11	0.090	0.41
	5 vs. 5	0.948	-0.02	0.308	0.24	0.534	0.19	0.627	0.08	0.937	-0.02	0.003	0.54	0.566	0.24	0.106	0.38
	6 vs. 6	0.619	-0.18	0.210	0.28	0.461	0.21	0.439	0.15	0.937	-0.02	0.000	0.67	0.617	0.22	0.624	0.17
	7 vs. 7	0.355	-0.26	0.426	0.19	0.335	0.28	0.337	0.21	0.927	-0.04	0.000	0.65	0.939	-0.02	0.194	0.31
	8 vs. 8	0.767	0.12	0.700	0.09	0.704	0.12	0.337	0.21	0.788	0.09	0.136	0.28	0.939	-0.02	0.848	-0.10

Journal of Experimental Biology • Supplementary information

Cosine similarity of the muscle weights

	compared cycles	anteroposterior								mediolateral							
		Weight acceptance		Propulsion		Early swing		Late swing		Weight acceptance		Propulsion		Early swing		Late swing	
		p-value	*d*	p-value	*d*	p-value	*d*	p-value	*d*	p-value	*d*	p-value	*d*	p-value	*d*	p-value	*d*
unpredictable	-3 vs. -2	-	-	-	-	-	-	-	-	-	-	-	-	-	-	-	-
	-3 vs. -1	0.941	0.02	0.587	-0.24	0.947	0.01	0.868	0.07	0.712	0.14	0.724	0.13	0.872	-0.15	0.938	-0.13
	-3 vs. 0	0.000	1.64	0.587	-0.22	0.000	1.79	0.000	2.05	0.110	0.47	0.846	-0.07	0.000	1.07	0.000	1.39
	-3 vs. 1	0.000	1.17	0.766	0.15	0.001	0.91	0.063	0.49	0.000	1.41	0.000	1.53	0.000	2.08	0.000	2.12
	-3 vs. 2	0.000	0.85	0.766	0.15	0.100	0.55	0.375	0.27	0.022	0.57	0.056	0.43	0.154	0.44	0.171	0.40
	-3 vs. 3	0.002	0.67	0.988	-0.01	0.864	0.16	0.257	0.32	0.000	0.82	0.056	0.43	0.110	0.49	0.071	0.52
	-3 vs. 4	0.157	0.36	0.901	-0.11	0.947	0.04	0.219	0.35	0.663	0.19	0.127	0.34	0.872	0.07	0.531	0.26
	-3 vs. 5	0.693	0.13	0.988	-0.04	0.783	0.28	0.868	0.09	0.045	0.51	0.888	-0.04	0.872	0.06	0.981	0.03
	-3 vs. 6	0.697	-0.12	0.587	0.22	0.864	-0.17	0.454	-0.25	0.591	-0.22	0.751	-0.12	0.872	0.12	0.938	0.12
	-3 vs. 7	0.680	-0.14	0.587	0.22	0.947	-0.03	0.210	-0.36	0.422	-0.29	0.138	-0.33	0.872	0.11	0.981	0.01
	-3 vs. 8	0.157	-0.35	0.587	0.24	0.947	-0.02	0.702	-0.17	0.712	-0.13	0.845	0.09	0.872	0.09	0.732	0.19
predictable	-3 vs. -2	-	-	-	-	-	-	-	-	-	-	-	-	-	-	-	-
	-3 vs. -1	0.697	-0.10	0.988	0.06	0.947	-0.05	0.938	0.02	0.862	-0.06	0.888	-0.03	0.990	0.00	0.981	0.05
	-3 vs. 0	0.030	0.47	0.901	0.11	0.000	1.34	0.000	1.28	0.460	0.26	0.191	0.29	0.000	1.67	0.000	1.48
	-3 vs. 1	0.000	0.73	0.034	0.54	0.100	0.47	0.210	0.38	0.862	0.06	0.061	0.40	0.746	0.26	0.171	0.40
	-3 vs. 2	0.916	-0.04	0.766	0.16	0.864	0.15	0.702	0.17	0.878	0.05	0.237	0.26	0.973	-0.02	0.981	-0.03
	-3 vs. 3	0.680	-0.12	0.988	0.07	0.864	0.14	0.484	0.22	0.712	0.14	0.335	0.22	0.872	0.12	0.981	0.02
	-3 vs. 4	0.916	-0.04	0.988	0.01	0.864	0.17	0.868	-0.05	0.881	0.04	0.846	0.07	0.872	-0.14	0.981	0.00
	-3 vs. 5	0.916	0.03	0.988	0.00	0.864	0.14	0.868	0.12	0.508	0.23	0.846	0.06	0.973	-0.02	0.938	-0.08
	-3 vs. 6	0.680	0.13	0.766	-0.16	0.947	-0.02	0.868	-0.07	0.712	-0.13	0.893	-0.02	0.872	0.10	0.981	-0.02
	-3 vs. 7	0.539	0.20	0.901	-0.11	0.947	0.03	0.868	-0.07	0.795	0.09	0.846	-0.07	0.872	-0.07	0.938	0.10
	-3 vs. 8	0.680	0.12	0.988	-0.02	0.864	-0.13	0.868	-0.07	0.684	0.16	0.864	0.05	0.872	0.07	0.938	-0.08
unpredictable vs. predictable	-3 vs. -3	-	-	-	-	-	-	-	-	-	-	-	-	-	-	-	-
	-2 vs. -2	0.397	-0.26	0.470	0.34	0.947	0.03	0.868	-0.05	0.422	-0.29	0.138	0.33	0.872	0.09	0.981	-0.03
	-1 vs. -1	0.680	-0.14	0.988	0.04	0.947	0.10	0.999	0.00	0.795	-0.09	0.027	0.49	0.872	-0.07	0.681	-0.21
	0 vs. 0	0.000	0.91	0.988	0.01	0.100	0.48	0.000	0.73	0.850	-0.08	0.888	-0.03	0.098	-0.51	0.938	-0.12
	1 vs. 1	0.680	0.17	0.988	-0.05	0.161	0.47	0.868	0.06	0.000	1.06	0.000	1.46	0.000	1.91	0.000	1.69
	2 vs. 2	0.002	0.63	0.470	0.33	0.205	0.43	0.868	0.05	0.508	0.24	0.028	0.49	0.093	0.55	0.171	0.40
	3 vs. 3	0.012	0.53	0.587	0.26	0.947	0.05	0.871	0.04	0.151	0.39	0.015	0.54	0.139	0.45	0.107	0.48
	4 vs. 4	0.680	0.14	0.609	0.21	0.947	-0.10	0.219	0.34	0.712	-0.14	0.005	0.61	0.686	0.29	0.647	0.23
	5 vs. 5	0.680	-0.17	0.571	0.29	0.864	0.17	0.868	-0.09	0.992	0.00	0.335	0.22	0.872	0.17	0.938	0.09
	6 vs. 6	0.947	-0.01	0.988	-0.04	0.864	0.17	0.868	0.12	0.634	-0.19	0.056	0.42	0.872	0.07	0.789	-0.17
	7 vs. 7	0.785	0.08	0.988	0.01	0.947	0.09	0.460	0.23	0.795	0.09	0.006	0.59	0.872	-0.09	0.972	0.07
	8 vs. 8	0.523	0.22	0.988	0.08	0.947	-0.09	0.868	0.06	0.992	0.01	0.191	0.29	0.872	0.07	0.365	-0.30

CoA

	compared cycles	anteroposterior								mediolateral							
		Weight acceptance		Propulsion		Early swing		Late swing		Weight acceptance		Propulsion		Early swing		Late swing	
		p-value	*d*	p-value	*d*	p-value	*d*	p-value	*d*	p-value	*d*	p-value	*d*	p-value	*d*	p-value	*d*
unpredictable	-3 vs. -2	0.218	-0.40	0.766	0.14	0.731	0.19	0.559	0.13	0.997	0.01	0.966	0.02	0.763	0.09	0.968	0.11
	-3 vs. -1	0.880	-0.05	0.925	-0.04	0.351	0.34	0.709	0.07	0.979	-0.18	0.569	0.14	0.763	0.09	0.995	0.02
	-3 vs. 0	0.000	-4.80	0.029	0.47	0.000	1.41	0.589	0.11	0.000	-4.10	0.475	-0.18	0.000	1.54	0.920	0.14
	-3 vs. 1	0.860	-0.13	0.368	-0.27	0.000	2.89	0.001	0.68	0.001	-0.76	0.000	1.87	0.000	1.84	0.000	0.90
	-3 vs. 2	0.860	0.12	0.011	0.53	0.731	0.19	0.001	0.64	0.997	-0.13	0.000	1.56	0.000	1.03	0.004	0.62
	-3 vs. 3	0.370	-0.30	0.182	0.35	0.661	0.25	0.110	0.36	0.997	0.11	0.000	1.02	0.152	0.39	0.968	0.10
	-3 vs. 4	0.584	-0.25	0.388	0.24	0.731	0.18	0.006	0.55	0.997	0.10	0.002	0.57	0.426	0.27	0.873	0.18
	-3 vs. 5	0.771	-0.20	0.197	0.33	0.731	0.15	0.074	0.39	0.997	0.00	0.328	0.23	0.763	0.11	0.873	0.18
	-3 vs. 6	0.860	0.12	0.780	0.12	0.825	0.07	0.233	0.28	0.997	0.03	0.416	0.20	0.122	0.44	0.995	0.00
	-3 vs. 7	0.880	0.07	0.925	0.04	0.731	0.14	0.133	0.32	0.937	-0.20	0.514	0.16	0.421	0.28	0.968	0.07
	-3 vs. 8	0.860	0.14	0.863	0.09	0.730	0.21	0.234	0.26	0.997	0.09	0.763	0.10	0.682	0.16	0.995	0.00
predictable	-3 vs. -2	0.880	-0.07	0.993	0.02	0.854	0.05	0.472	0.16	0.997	-0.04	0.987	0.00	0.044	0.52	0.995	-0.01
	-3 vs. -1	0.880	-0.06	0.925	0.04	0.730	0.19	0.445	0.17	0.997	0.03	0.514	0.16	0.682	0.14	0.968	0.10
	-3 vs. 0	0.000	-1.45	0.002	-0.63	0.006	0.60	0.589	0.11	0.000	-1.66	0.328	-0.22	0.000	1.30	0.873	0.18
	-3 vs. 1	0.281	-0.34	0.820	-0.10	0.000	1.38	0.445	0.17	0.997	0.00	0.000	0.81	0.218	0.35	0.000	0.71
	-3 vs. 2	0.880	-0.07	0.993	0.00	0.351	0.32	0.234	0.25	0.997	0.00	0.001	0.56	0.338	0.28	0.873	0.20
	-3 vs. 3	0.880	-0.07	0.921	0.06	0.731	0.10	0.234	0.25	0.822	-0.22	0.951	-0.04	0.763	-0.08	0.873	0.16
	-3 vs. 4	0.860	-0.11	0.993	0.00	0.731	-0.12	0.241	0.25	0.997	0.00	0.951	-0.03	0.587	0.19	0.995	0.03
	-3 vs. 5	0.880	-0.08	0.925	-0.04	0.731	0.17	0.133	0.32	0.997	-0.05	0.987	-0.01	0.548	0.21	0.920	0.12
	-3 vs. 6	0.860	-0.15	0.780	-0.12	0.825	-0.07	0.472	0.16	0.997	0.02	0.569	-0.13	0.869	0.04	0.995	-0.02
	-3 vs. 7	0.860	-0.14	0.921	-0.07	0.886	0.03	0.445	0.17	0.997	0.04	0.328	-0.23	0.256	0.32	0.995	0.02
	-3 vs. 8	0.860	-0.11	0.463	-0.21	0.731	-0.11	0.445	0.18	0.997	0.00	0.475	-0.17	0.940	-0.02	0.968	0.07
unpredictable vs. predictable	-3 vs. -3	0.700	-0.21	0.993	0.01	0.565	-0.26	0.876	0.03	0.997	-0.10	0.951	0.03	0.763	-0.08	0.968	0.08
	-2 vs. -2	0.032	-0.54	0.780	0.13	0.731	-0.12	0.974	0.00	0.997	-0.05	0.893	0.06	0.049	-0.51	0.873	0.20
	-1 vs. -1	0.700	-0.21	0.921	-0.07	0.731	-0.11	0.709	-0.07	0.473	-0.32	0.972	0.02	0.731	-0.13	0.995	0.00
	0 vs. 0	0.000	-3.56	0.000	1.11	0.012	0.55	0.876	0.03	0.000	-2.54	0.878	0.07	0.641	0.16	0.995	0.04
	1 vs. 1	0.999	0.00	0.761	-0.16	0.000	1.25	0.011	0.54	0.000	-0.86	0.000	1.09	0.000	1.41	0.873	0.26
	2 vs. 2	0.952	-0.02	0.011	0.53	0.255	-0.39	0.054	0.41	0.822	-0.23	0.000	1.03	0.005	0.67	0.030	0.50
	3 vs. 3	0.115	-0.44	0.281	0.29	0.731	-0.11	0.526	0.14	0.822	0.23	0.000	1.09	0.141	0.39	0.995	0.02
	4 vs. 4	0.278	-0.35	0.388	0.24	0.854	0.04	0.133	0.33	0.997	0.00	0.000	0.64	0.998	0.00	0.873	0.23
	5 vs. 5	0.330	-0.33	0.132	0.37	0.514	-0.28	0.606	0.10	0.997	-0.05	0.232	0.27	0.652	-0.17	0.920	0.13
	6 vs. 6	0.880	0.06	0.374	0.25	0.731	-0.12	0.485	0.15	0.997	-0.09	0.067	0.37	0.288	0.32	0.968	0.09
	7 vs. 7	0.999	0.00	0.798	0.11	0.731	-0.15	0.445	0.18	0.396	-0.34	0.028	0.42	0.763	-0.12	0.920	0.13
	8 vs. 8	0.913	0.04	0.248	0.30	0.828	0.06	0.589	0.12	0.997	-0.01	0.162	0.30	0.763	0.10	0.995	0.01

FWHM

	compared cycles	anteroposterior								mediolateral							
		Weight acceptance		Propulsion		Early swing		Late swing		Weight acceptance		Propulsion		Early swing		Late swing	
		p-value	*d*	p-value	*d*	p-value	*d*	p-value	*d*	p-value	*d*	p-value	*d*	p-value	*d*	p-value	*d*
unpredictable	-3 vs. -2	0.866	-0.13	0.991	-0.04	0.611	-0.24	0.954	-0.04	0.803	-0.06	0.705	0.18	0.678	0.21	0.980	0.10
	-3 vs. -1	0.890	-0.06	0.954	0.10	0.957	0.05	0.915	0.09	0.509	0.18	0.751	-0.15	0.728	0.12	0.801	0.21
	-3 vs. 0	0.000	-1.93	0.292	-0.32	0.116	-0.41	0.386	-0.33	0.000	-1.31	0.000	-1.14	0.685	-0.13	0.340	-0.36
	-3 vs. 1	0.213	-0.38	0.004	0.65	0.957	-0.02	0.013	-0.62	0.012	-0.62	0.950	0.03	0.678	-0.17	0.000	-1.18
	-3 vs. 2	0.866	-0.11	0.954	-0.14	0.957	0.02	0.302	-0.37	0.240	0.34	0.271	-0.30	0.678	-0.17	0.980	-0.04
	-3 vs. 3	0.866	-0.16	0.954	-0.10	0.957	-0.09	0.954	-0.02	0.401	-0.25	0.116	-0.37	0.938	0.02	0.340	-0.36
	-3 vs. 4	0.866	0.12	0.954	-0.12	0.839	-0.12	0.436	-0.29	0.390	0.29	0.634	-0.21	0.938	0.02	0.980	-0.11
	-3 vs. 5	0.890	0.06	0.692	-0.22	0.725	-0.15	0.954	0.01	0.625	0.13	0.836	-0.08	0.678	0.22	0.980	0.05
	-3 vs. 6	0.947	0.03	0.991	0.00	0.957	-0.02	0.954	-0.01	0.915	-0.03	0.836	-0.08	0.685	0.16	0.980	0.01
	-3 vs. 7	0.890	0.09	0.991	0.02	0.957	-0.08	0.954	0.03	0.524	-0.17	0.818	0.12	0.678	0.24	0.980	0.11
	-3 vs. 8	0.866	-0.13	0.991	-0.02	0.725	-0.18	0.954	0.06	0.524	0.17	0.976	-0.01	0.678	0.20	0.980	0.02
predictable	-3 vs. -2	0.998	0.00	0.799	0.20	0.196	-0.34	0.816	-0.18	0.401	0.22	0.751	0.15	0.678	-0.23	0.985	0.00
	-3 vs. -1	0.947	0.02	0.991	0.01	0.225	-0.32	0.915	-0.14	0.390	0.25	0.976	0.01	0.678	-0.21	0.937	0.17
	-3 vs. 0	0.000	-1.02	0.092	0.40	0.000	-0.94	0.010	-0.61	0.000	-1.11	0.020	-0.47	0.685	-0.13	0.000	-1.10
	-3 vs. 1	0.010	-0.55	0.423	0.28	0.725	0.14	0.145	-0.44	0.870	0.04	0.002	-0.58	0.414	-0.46	0.396	-0.30
	-3 vs. 2	0.890	-0.08	0.991	0.04	0.080	-0.45	0.915	-0.11	0.625	0.12	0.060	-0.40	0.938	0.03	0.697	-0.22
	-3 vs. 3	0.694	-0.27	0.991	0.05	0.370	-0.26	0.954	0.04	0.401	0.21	0.836	0.09	0.988	0.00	0.980	-0.02
	-3 vs. 4	0.890	-0.06	0.991	0.07	0.626	-0.20	0.954	-0.05	0.629	0.11	0.651	0.19	0.678	-0.18	0.980	0.13
	-3 vs. 5	0.866	0.13	0.954	0.09	0.725	-0.13	0.816	-0.18	0.625	0.11	0.818	0.12	0.856	-0.06	0.980	0.02
	-3 vs. 6	0.866	-0.14	0.954	0.10	0.957	-0.04	0.954	-0.05	0.092	0.43	0.950	0.03	0.685	-0.14	0.980	0.07
	-3 vs. 7	0.890	-0.08	0.954	0.08	0.340	-0.28	0.915	-0.12	0.122	0.39	0.950	0.04	0.685	-0.14	0.980	0.08
	-3 vs. 8	0.987	0.01	0.954	0.13	0.957	-0.05	0.915	0.10	0.390	0.24	0.950	0.03	0.728	-0.09	0.980	0.09
unpredictable vs. predictable	-3 vs. -3	0.943	-0.04	0.864	0.18	0.957	-0.03	0.954	-0.06	0.441	0.20	0.950	0.05	0.728	-0.10	0.980	-0.02
	-2 vs. -2	0.866	-0.17	0.991	-0.05	0.957	0.07	0.915	0.09	0.743	-0.08	0.836	0.09	0.678	0.34	0.980	0.09
	-1 vs. -1	0.866	-0.12	0.423	0.27	0.225	0.34	0.824	0.17	0.620	0.13	0.818	-0.11	0.678	0.23	0.980	0.02
	0 vs. 0	0.000	-0.95	0.011	-0.55	0.028	0.50	0.634	0.23	0.979	0.01	0.002	-0.62	0.728	-0.10	0.003	0.72
	1 vs. 1	0.866	0.13	0.013	0.55	0.725	-0.18	0.688	-0.24	0.092	-0.46	0.002	0.66	0.678	0.19	0.000	-0.90
	2 vs. 2	0.890	-0.07	0.991	0.00	0.116	0.44	0.411	-0.31	0.115	0.43	0.751	0.15	0.678	-0.31	0.980	0.16
	3 vs. 3	0.890	0.07	0.991	0.03	0.725	0.15	0.915	-0.11	0.390	-0.26	0.060	-0.41	0.754	-0.08	0.340	-0.35
	4 vs. 4	0.866	0.14	0.991	-0.01	0.957	0.05	0.436	-0.30	0.153	0.38	0.116	-0.35	0.728	0.10	0.599	-0.26
	5 vs. 5	0.866	-0.11	0.954	-0.13	0.957	-0.04	0.915	0.14	0.401	0.22	0.751	-0.15	0.678	0.18	0.980	0.01
	6 vs. 6	0.866	0.13	0.954	0.09	0.978	0.00	0.954	-0.02	0.390	-0.25	0.922	-0.06	0.678	0.19	0.980	-0.07
	7 vs. 7	0.866	0.13	0.954	0.12	0.725	0.17	0.915	0.09	0.172	-0.36	0.818	0.12	0.678	0.28	0.980	0.01
	8 vs. 8	0.866	-0.18	0.991	0.03	0.725	-0.16	0.915	-0.09	0.608	0.14	0.976	0.00	0.678	0.19	0.980	-0.10

Journal of Experimental Biology • Supplementary information

	Compared cycles	Gait parameter															
		Cadence				Stance time				Swing time				Duty factor			
		anteroposterior		mediolateral		anteroposterior		mediolateral		anteroposterior		mediolateral		anteroposterior		mediolateral	
		p-value	*d*	p-value	*d*	p-value	*d*	p-value	*d*	p-value	*d*	p-value	*d*	p-value	*d*	p-value	*d*
unpredictable	-3 vs. -2	0.998	0.01	0.993	-0.01	0.954	0.02	0.973	0.00	0.860	-0.07	0.951	0.04	0.876	0.07	0.910	-0.03
	-3 vs. -1	0.998	-0.01	0.993	0.00	0.954	0.01	0.893	-0.03	0.887	0.04	0.884	0.08	0.876	-0.02	0.881	-0.09
	-3 vs. 0	0.000	-1.67	0.000	-1.52	0.000	1.15	0.052	0.25	0.000	3.05	0.000	3.74	0.000	-1.53	0.000	-3.40
	-3 vs. 1	0.000	-3.27	0.000	-4.21	0.000	2.77	0.000	3.77	0.000	2.24	0.000	1.95	0.000	1.37	0.000	2.05
	-3 vs. 2	0.000	-0.96	0.000	-1.96	0.000	1.29	0.000	2.45	0.043	0.31	0.002	0.48	0.000	1.14	0.000	2.02
	-3 vs. 3	0.001	-0.50	0.000	-1.15	0.000	0.62	0.000	1.43	0.026	0.34	0.001	0.52	0.091	0.37	0.000	0.88
	-3 vs. 4	0.012	-0.41	0.000	-0.67	0.000	0.54	0.000	0.89	0.281	0.20	0.127	0.29	0.073	0.40	0.001	0.55
	-3 vs. 5	0.116	-0.28	0.004	-0.44	0.005	0.37	0.000	0.62	0.482	0.15	0.409	0.18	0.448	0.26	0.022	0.38
	-3 vs. 6	0.258	-0.23	0.071	-0.30	0.077	0.25	0.002	0.39	0.230	0.22	0.318	0.21	0.964	0.08	0.417	0.15
	-3 vs. 7	0.328	-0.20	0.129	-0.26	0.093	0.24	0.014	0.31	0.531	0.14	0.280	0.23	0.876	0.13	0.695	0.07
	-3 vs. 8	0.267	-0.22	0.366	-0.17	0.089	0.24	0.161	0.19	0.406	0.17	0.339	0.20	0.876	0.13	0.910	-0.01
predictable	-3 vs. -2	0.998	0.01	0.935	-0.03	0.954	-0.01	0.553	0.09	0.970	0.01	0.949	-0.06	0.964	-0.02	0.658	0.12
	-3 vs. -1	0.826	-0.07	0.711	-0.09	0.664	0.09	0.228	0.15	0.887	0.04	0.951	-0.01	0.876	0.06	0.910	0.14
	-3 vs. 0	0.000	-1.52	0.000	-0.92	0.000	1.23	0.000	0.45	0.000	2.28	0.000	1.73	0.000	-0.71	0.000	-1.27
	-3 vs. 1	0.000	-0.86	0.000	-0.73	0.000	1.03	0.000	1.03	0.230	0.22	0.658	0.12	0.000	1.03	0.000	0.84
	-3 vs. 2	0.816	-0.09	0.216	-0.21	0.706	0.07	0.000	0.41	0.560	0.13	0.643	-0.13	0.964	-0.01	0.049	0.46
	-3 vs. 3	0.826	-0.07	0.711	-0.09	0.363	0.14	0.177	0.17	0.963	-0.01	0.951	-0.02	0.472	0.15	0.900	0.16
	-3 vs. 4	0.998	-0.02	0.895	-0.05	0.954	-0.02	0.454	0.11	0.655	0.11	0.951	-0.02	0.876	-0.09	0.990	0.11
	-3 vs. 5	0.980	-0.03	0.895	-0.05	0.901	0.04	0.890	0.03	0.887	0.05	0.690	0.10	0.964	0.00	0.431	-0.06
	-3 vs. 6	0.998	0.00	0.969	-0.02	0.954	0.02	0.849	0.04	0.887	-0.05	0.951	-0.01	0.876	0.05	0.850	0.05
	-3 vs. 7	0.998	0.00	0.895	-0.05	0.954	-0.01	0.915	0.02	0.887	0.03	0.658	0.12	0.964	-0.03	0.417	-0.08
	-3 vs. 8	0.950	0.04	0.993	-0.01	0.706	-0.07	0.973	0.01	0.983	0.00	0.951	0.02	0.876	-0.07	0.658	0.00
unpredictable vs. predictable	-3 vs. -3	0.826	0.07	0.969	0.02	0.664	-0.08	0.943	-0.02	0.860	-0.07	0.885	-0.07	0.964	-0.02	0.900	0.05
	-2 vs. -2	0.826	0.07	0.898	0.05	0.808	-0.05	0.462	-0.11	0.482	-0.15	0.951	0.03	0.876	0.06	0.695	-0.10
	-1 vs. -1	0.634	0.12	0.653	0.11	0.286	-0.16	0.128	-0.20	0.878	-0.06	0.951	0.02	0.807	-0.11	0.417	-0.19
	0 vs. 0	0.826	-0.08	0.000	-0.58	0.268	-0.17	0.110	-0.21	0.000	0.70	0.000	1.93	0.000	-0.85	0.000	-2.08
	1 vs. 1	0.000	-2.34	0.000	-3.46	0.000	1.66	0.000	2.73	0.000	1.95	0.000	1.77	0.079	0.32	0.000	1.26
	2 vs. 2	0.000	-0.80	0.000	-1.73	0.000	1.14	0.000	2.02	0.636	0.12	0.000	0.54	0.000	1.12	0.000	1.60
	3 vs. 3	0.030	-0.36	0.000	-1.04	0.002	0.40	0.000	1.24	0.077	0.28	0.002	0.46	0.413	0.20	0.000	0.77
	4 vs. 4	0.061	-0.32	0.000	-0.60	0.000	0.48	0.000	0.77	0.933	0.02	0.250	0.24	0.004	0.46	0.005	0.49
	5 vs. 5	0.383	-0.18	0.016	-0.37	0.084	0.25	0.000	0.57	0.887	0.03	0.951	0.01	0.284	0.23	0.005	0.48
	6 vs. 6	0.470	-0.16	0.124	-0.26	0.313	0.15	0.008	0.33	0.281	0.20	0.525	0.16	0.989	0.00	0.523	0.15
	7 vs. 7	0.608	-0.13	0.316	-0.18	0.290	0.16	0.032	0.27	0.887	0.03	0.951	0.03	0.646	0.14	0.417	0.19
	8 vs. 8	0.335	-0.20	0.467	-0.14	0.102	0.23	0.202	0.17	0.708	0.10	0.658	0.12	0.472	0.17	0.910	0.04

Journal of Experimental Biology • Supplementary information
